# Short hairpin RNA targeting insulin-like growth factor binding protein-3 restores the bioavailability of insulin-like growth factor-1 in diabetic rats

**DOI:** 10.1590/S1677-5538.IBJU.2014.0416

**Published:** 2016

**Authors:** Zhang-Yan Zhou, Guang-Jun Zhong, Shao-Ping Cheng, Hui Huang, Jing Wang, Hui Pan, Chang-Mao Liu, Cheng Xing, Ya-Ling Sun, Rong-Hua Liu, Fei-Li

**Affiliations:** Department of Urology, First Affiliated Hospital of Yangtze University, Jingzhou, HuBei, China

**Keywords:** Insulin-like growth factor binding protein-3, insulin-like growth factor-1, erectile dysfunction

## Abstract

**Purpose:**

To investigate whether intracavernosal injection of short hairpin RNA for IGFBP-3 could improve erectile function in streptozotocin-induced diabetic rats.

**Materials and methods:**

After 12 weeks of IGFBP-3 short hairpin RNA injection treatment, intracavernous pressure responses to electrical stimulation of cavernous nerves were evaluated. The expression of IGFBP-3 and IGF-1 at mRNA and protein levels were detected by quantitative real-time PCR analysis and Western blot, respectively. The concentration of cavernous cyclic guanosine monophosphate was detected by enzyme-linked immunosorbent assay.

**Results:**

At 12 weeks after intracavernous administration of IGFBP-3 shRNA, the cavernosal pressure was significantly increased in response to the cavernous nerves stimulation compared to the diabetic group (P<0.05). Cavernous IGFBP-3 expression at both mRNA and protein levels was significantly inhibited. At the same time, cavernous IGF-1 expression was significantly increased in the IGFBP-3 shRNA treatment group compared to the diabetic group (P<0.01). Cavernous cyclic guanosine monophosphate concentration was significantly increased in the IGFBP-3 shRNA treatment group compared to the diabetic group (P<0.01).

**Conclusions:**

Gene transfer of IGFBP-3 shRNA could improve erectile function via the restoration of cavernous IGF-1 bioavailability and an increase of cavernous cGMP concentration in the pathogenesis of erectile dysfunction in streptozotocin-induced diabetic rats.

## INTRODUCTION

Erectile dysfunction (ED) has been increasingly recognized as a public health problem, estimated to affect approximately 150 million men worldwide (1). Current research on erectile physiology has focused on the pathogenesis of ED and provided convincing evidence that diabetes is one of the most prevalent causes of ED (2).

Insulin-like growth factor binding protein-3 (IGFBP-3) is a member of the growth factor family (3). As an important growth factor, IGFBP-3 has been reported that it is highly correlated with erectile function in diabetic rat model and the increased expression of IGFBP-3 could elicit ED in patients with diabetes mellitus (DM) (2). In some previous studies, it has been also reported that the increased expression of IGFBP-3 is associated with ED in hypertensive and streptozotocin (STZ)-induced diabetic rats (4, 5). IGFBP-3 may limit the bioavailability of insulin-like growth factor-1 (IGF-1), while IGF-1 is associated with ED in rat model (6, 7).

Since short hairpin RNA (shRNA) emerges as a technology to silence gene expression by inhibiting mRNA translation and/or inducing its degradation (8), therefore, in this study we employed shRNA technology to investigate whether intracavernosal injection of shRNA for IGFBP-3 can ameliorate diabetes-related ED.

## MATERIAL AND METHODS

### IGFBP-3 shRNA Constructs

To design IGFBP-3 shRNA construct, the rat IGFBP-3 gene sequence (GI:M31837) was analyzed for a potential siRNA target using the web-based siRNA target finder and design tool provided on the Ambion website (Ambion, Inc., Austin, TX). Double-stranded siRNAs (nucleotide position: 611) were transcribed “in vitro” using the Silencer siRNA construction kit (Ambion) following the manufacturer’s instructions. The inhibitory siRNA (5′-GCGCTACAAAGTTGACTATGA-3′, nucleotide position 611) was then cloned into the pGPU6/GFP/Neo plasmid vector (2nd version, Ambion) as a short hairpin DNA sequence (5′ sense strand:5′-CACCGCGCTACAAAGTTGACTATGATTCAAGAGATCATAGTCAACTTTGTAGCGCTTTTTTG-3′) according to the manufacturer’s instructions. The pGPU6/GFP/Neo-IGFBP-3 shRNA plasmid was purified using Endo-free Maxi kit (Qiagen, Valencia, CA). Plasmids were quantitated by spectrophotometry and prepared in 0.9% saline solution at a concentration of 1.0μg/μL.

### Experimental Animals

Twenty seven adult male Wistar rats (Grade SPF, 3-month-old, weight 310-330g, certificate No. scxk (E) 2008-0005) were obtained from Hubei Research Center of Laboratory Animal (Wuhan, China). The rats were randomly divided into three groups with nine rats in each group. Group 1 included 9 normal control rats that received i.p. injection of citrate buffer (100mmol/L citric acid and 200mmol/L disodium phosphate, pH 7.0). The other 18 rats received i.p. injection of STZ at a dose of 65mg/kg. Rats were considered diabetic if blood glucose level was greater than 200mg/dL (Table-1). Animals received 60μL citrate buffer (group 1 and 2) and 60μL IGFBP-3 shRNA (10μL/kg) (group 3) into the corpus cavernosum at 12 weeks after STZ induction, respectively. Half of the dose was administered in each crus. During intracavernosal injection, a constriction band was applied at the base of the penis, and the needle was left in place for 5 min to allow the medication (lipofectamine-plasmid complex) to diffuse throughout the cavernous space (7). The animal experiments were approved by Wuhan University Animal Care and Use Committee.

### Measurement of Erectile Responses

Erectile function was assessed by measuring intracavernous pressure (ICP) following electrostimulation of the cavernous nerves at 12 weeks after IGFBP-3 shRNA administration, as previously described (7). Mean arterial pressure (MAP) and ICP lines were connected to a pressure transducer, which was then connected via a transducer amplifier to a data acquisition board (RM6240, Chengdu Instrument, Chengdu, China). Electrical stimulation of the cavernous nerves (1ms pulse, 60s, 15Hz, 2.5V) was performed. The ratio of maximal ICP-to-MAP (ICP/MAP) and total ICP (the area under curve) were recorded for each rat. After measurement of the erectile responses, all rats were killed with an i.p. overdose of pentobarbital (80mg/kg) and the penile shaft was removed for other analysis.

### Quantitative Real-Time PCR Analysis

Total RNA was extracted from rat penile samples using TRIzol^TM^ reagent (Invitrogen, Merelbeke, Belgium) according to the manufacturer’s protocol and re-suspended in RNAse-free water. Total RNA was stored at-80ºC until analysis of IGFBP-3 and IGF-1mRNA levels by MyiQ single color real-time PCR detection system (Bio-Rad laboratories, Hercules, CA) with following primers: IGFBP-3, forward 5’-AGCCGTCTCCTGGAAACACC-3’ and reverse 5’-CCCGCTTTCTGCCTTTGG-3’; IGF-1, forward 5’-GACATGCCCAAGACCCAGAAGGA-3’ and reverse 5’-CGGTGGCATGTCACTCTTCACTC-3’.

Quantitative mRNA measurements were performed in triplicate and normalized to an internal control of GAPDH. After the PCR program, data were analyzed with the ABI 7300 SDS software (Applied Biosystems, Foster City, CA, USA).

### Western Blot Analysis

As previously described (7), the rat penile samples were lysed in RIPA buffer (1xTBS, 0.1% SDS, 0.004% sodium azide, 1% Nonidet P-40, 10μL/ml PMSF, 10μL/mL protease inhibitor cocktail, 0.5% sodium deoxycholate, 10μL/mL sodium orthovanadate). Protein concentration was measured using the Coomassie Plus Protein Assay Reagent™ (Pierce Biotechnology, Rockford, IL). Equal quantities (30μg) of lysates were separated on 10% sodium dodecyl sulfate gradient polyacrylamide gels and electroblotted onto PVDF membranes (Bio-Rad, Hercules, CA, USA). Then the membranes were blocked and incubated with rabbit anti-IGFBP-3 antibody (Sc-9028) or anti-IGF-1 (Sc-9013) antibody at 1:1000 dilutions, respectively (Santa Cruz Biotechnology, Santa Cruz, CA, USA). Chemiluminescence was detected using ECL Western blotting detection reagents (Amersham, Buckinghamshire, UK). The IGFBP-3 protein expression was visualized by densitometry using the Mivnt Image analysis system (Shanghai Institute of Optical Instruments, Shanghai, China).

### Enzyme-linked Immunosorbent Assay

Cyclic guanosine monophosphate (cGMP) concentration in the lysate of rat penile tissue was determined using an enzyme-linked immunosorbent assay (ELISA) cGMP detection kit (R&D Systems, Minneopolis, MN, USA) following the manufacturer’s instruction.

### Statistical analysis

Data were expressed as mean±SD. Differences were considered significant at the level of p<0.05 using two-tailed unpaired t test.

## RESULTS

### IGFBP-3 shRNA Improves Erectile Function in Diabetic Rats

**Figure 1:**
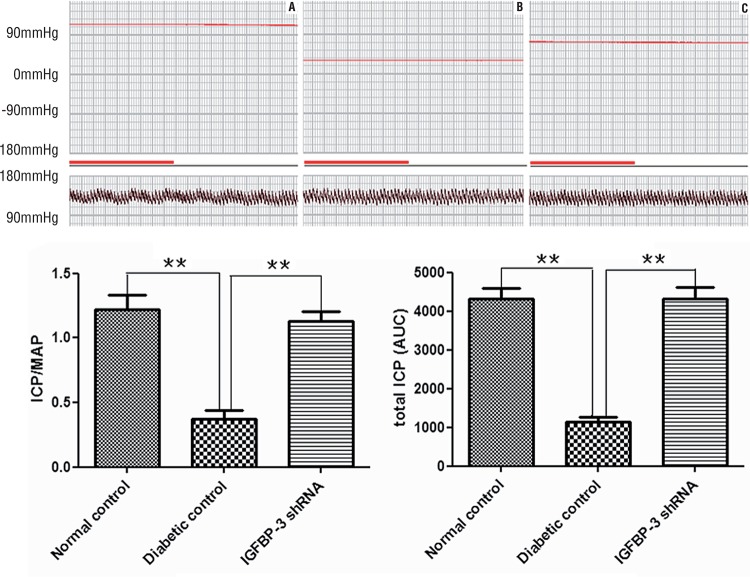
IGFBP-3 shRNA improves erectile function in diabetic rats.

We measured ICP/MAP ratio and total ICP (the area under curve) during electrostimulation of the cavernous nerves in three groups at 12 weeks after intracavernous administration of IGFBP-3 shRNA. The representative ICP tracing in response to electrostimulation of the cavernous nerves is shown in Figure-1 (upper panel). Electrostimulation in IGFBP-3 shRNA treatment group elicited significantly increased ICP/MAP ratio (Figure-1, lower: left) and total ICP (Figure-1, lower: right) compared to those in diabetic control group (P<0.01, respectively). These data suggest that IGFBP-3 shRNA treatment improved erectile function in diabetic rats.

Representative ICP responses to cavernous nerves electrical stimulation at 12 weeks after intracavernous administration of IGFBP-3 shRNA in normal control **(A)**, diabetic control **(B)** and IGFBP-3 shRNA treatment **(C)** (n=9). Bold line indicated 1 min of electrical stimulation to cavernous nerve. The data of ICP/MAP (lower, left) and total ICP (the area under curve) (lower, right) were collected from nine rats in each group. * P<0.05, compared to diabetic control, **: P<0.01, compared to diabetic control. **MAP =** mean arterial pressure; **ICP** = intracavernous pressure.

### IGFBP-3 shRNA Inhibits IGFBP-3 Expression and Improves IGF-1 bioavailability in Rat Cavernous Tissue

**Figures 2 and 3:**
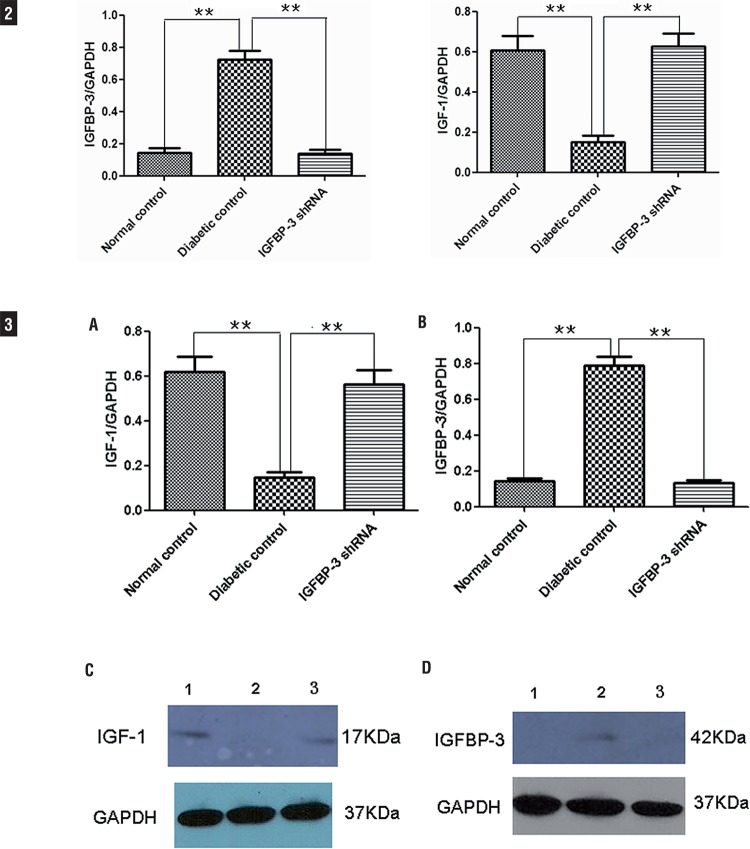
IGFBP-3 shRNA Inhibits IGFBP-3 Expression and Improves IGF-1 bioavailability in Rat Cavernous Tissue.

To confirm that inhibition of IGFBP-3 expression contributes to IGFBP-3 shRNA mediated improvement in erectile function in diabetic rats, we examined IGFBP-3 expression level in rat cavernous tissues at 12 weeks after intracavernous administration of IGFBP-3 shRNA. Real-time qPCR analysis using GAPDH as a housekeeping gene showed that cavernous IGFBP-3 mRNA level in IGFBP-3 shRNA treatment group was significantly lower than in diabetic control group (P<0.01, Figure-2). In addition, as shown in Figure-2, it was showed that cavernous IGF-1 mRNA level in IGFBP-3 shRNA treatment group was significantly higher than in diabetic control group (P<0.01, Figure-2). Accordingly, Western blot analysis showed hat cavernous IGFBP-3 protein level in the IGFBP-3 shRNA treatment group was significantly lower than in the diabetic control group (P<0.01, Figure 3 B and D), while it was showed that cavernous IGF-1 protein level in IGFBP-3 shRNA treatment group was significantly higher than in diabetic control group (P<0.01, Figures 3 A and C). These results were consistent with the results of real-time qPCR analysis and proved that IGFBP-3 shRNA inhibits IGFBP-3 expression and improves IGF-1 bioavailability in rat cavernous tissue.

Quantitative real time-PCR and Western blot analyses of IGFBP-3 and IGF-1 mRNA and protein levels in rat cavernous tissue at 12 weeks after intracavernous administration of IGFBP-3 shRNA. The data were collected from nine rats in each group. ** P<0.01, compared to diabetic control.

### IGFBP-3 shRNA Increases cGMP Concentration in Rat Cavernous Tissue

**Figure 4:**
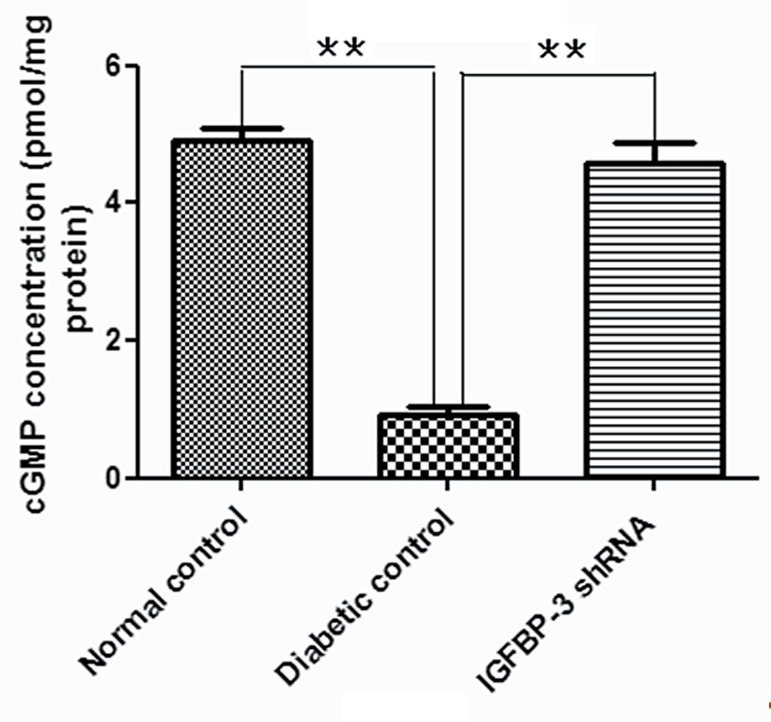
cGMP Concentrations in Rat Cavernous Tissue.

At 12 weeks after intracavernous administration of IGFBP-3 shRNA cavernous cGMP concentration was significantly increased in IGFBP-3 shRNA treatment group compared to that in diabetic control group (P<0.01, Figure-4).

cGMP concentrations in rat penile tissue at 12 weeks after intracavernous administration of IGFBP-3 shRNA were determined by ELISA. The data were collected from nine rats in each group.

** p<0.01, compared to diabetic control.

## DISCUSSION

In the current study we demonstrated that intracavernosal injection of IGFBP-3 shRNA could improve erectile responses in STZ-induced diabetic rats. We also presented evidence that improved erectile responses derived from IGFBP-3 shRNA might be caused by the restoration of IGF-1 activities and an increase of cGMP concentration in rat penile tissue.

A previous finding shows that IGFBP-3 can prevent its interaction with membrane receptors as a potent inhibitor of cell growth by virtue of its ability to bind insulin-like growth factor (IGF) with high affinity, including IGF-1 (2), while IGF-1 plays a key role in the regeneration of nitric oxide synthase (NOS)-containing nerve fibers in the dorsal and intracavernosal nerves (7). The nitric oxide (NO) derived from these nerves can cause vasodilatation, increase blood flow, smooth muscle relaxation, and penile erection (9, 10). Previously, it has been reported that there is a downregulation of IGF-1 protein expression in penile cavernosum of diabetic rats with ED (11). Here, we confirmed that cavernous IGFBP-3 mRNA and protein levels were significantly lower in the IGFBP-3 shRNA treatment group than those in the diabetic control group (p<0.01). The improved erectile responses might be caused by an increase of IGF-1 bioavailability after intracavernosal injection of IGFBP-3 shRNA. However, this is in contrast with published results in which no variations of IGF-1 mRNA is reported in rats after STZ diabetes induction (2).

On the other hand, evidence reported so far suggests that ED in diabetic animals and patients is mainly caused by the impairment of NO–cGMP signaling activities (10). The major subcellular mechanism by which erection occurs involves NO-induced activation of soluble guanylyl cyclase (sGC) and increased cGMP concentration (12, 13). The subsequent activation of cGMP-dependent protein kinase 1 (PKG1) causes smooth muscle relaxation through the inhibition of calcium flux (14). A decrease of cavernous cGMP concentration is associated with an impairment of cavernosal smooth muscle relaxation with a resultant decrease in erectile function (10, 15). As expected, agents that may increase cGMP concentration should enhance the relaxation of cavernosal smooth muscle and thereby can be applied for treatment of ED. In this study we measured cGMP concentration in rat penile tissue and found that cGMP concentration was significantly increased in the IGFBP-3 shRNA treatment group than that in the diabetic control group, indicating that the NO-cGMP signaling is restored by gene transfer of IGFBP-3 shRNA in diabetic rats. Taken together, these data strongly suggest that IGFBP-3 shRNA could antagonize downregulation of the NO-cGMP signaling and ameliorate diabetes-related ED. These results were also in agreement with physiological studies showing that gene transfer of shRNA-IGFBP-3 could improve erectile function in STZ-induced DM rats by an increase in the cyclic guanosine monophosphate concentration in cavernous tissue (5). However, long-term efficacy and safety studies of the current procedure are needed in future.

## CONCLUSIONS

Here we provide two lines of evidence that gene transfer of IGFBP-3 shRNA could improve erectile function via the restoration of cavernous IGF-1 bioavailability and an increase of cavernous cGMP concentration in the pathogenesis of erectile dysfunction in STZ-induced diabetic rats. However, several concerns should be addressed in future investigations to pave the way for its translation into clinical practice. For example, we need to evaluate the long-term efficacy and safety of this procedure.
